# Gold Nanoparticles for Modulating Neuronal Behavior

**DOI:** 10.3390/nano7040092

**Published:** 2017-04-24

**Authors:** Chiara Paviolo, Paul R. Stoddart

**Affiliations:** 1 LP2N-Institut d’Optique & CNRS, University of Bordeaux, 33400 Talence, France; 2 ARC Training Centre in Biodevices, Faculty of Science, Engineering and Technology, Swinburne University of Technology, Hawthorn, P.O. Box 218, Victoria 3122, Australia; pstoddart@swin.edu.au

**Keywords:** gold nanoparticles, plasmonics, photothermal effects, neuronal cell behavior

## Abstract

Understanding the detailed functioning and pathophysiology of the brain and the nervous system continues to challenge the scientific community, particularly in terms of scaling up techniques for monitoring and interfacing with complex 3D networks. Nanotechnology has the potential to support this scaling up, where the eventual goal would be to address individual nerve cells within functional units of both the central and peripheral nervous system. Gold nanoparticles provide a variety of physical and chemical properties that have attracted attention as a light-activated nanoscale neuronal interface. This review provides a critical overview of the photothermal and photomechanical properties of chemically functionalized gold nanoparticles that have been exploited to trigger a range of biological responses in neuronal tissues, including modulation of electrical activity and nerve regeneration. The prospects and challenges for further development are also discussed.

## 1. Introduction

The nervous system is essential to the functional transmission and processing of information within the human body. It consists of two main parts: the central nervous system (CNS), which includes the brain and spinal cord, and the peripheral nervous system (PNS) that comprises all other neural tissues in the body [1]. The basic unit of the nervous system is the neuron, a sophisticated biological machine capable of receiving and sending electrical signals on millisecond time-scales [2]. The complex electrical network that neurons form throughout the body constitutes the key mechanism for organ communication and for maintaining all physiological functions. Under pathological conditions, this pathway can be partially or totally disrupted, resulting in the loss of electrical transmission. Clinical therapies to restore the damaged neuronal network range from axonal gap connections (<25 mm) [3] to neural prostheses and neural interfaces for non-treatable conditions (e.g., neuro-degenerative diseases or spinal cord injuries). In this context, nanomaterials are expected to introduce new opportunities and provide improvements in current cell-based or immunological therapies [1]. Due to their small size, nanotechnology-based devices can interact with biological systems at the molecular level, with a high degree of spatial and temporal specificity. They can penetrate the blood-brain barrier and deliver specific therapeutic agents, probes, or biological materials to targeted cells and tissues [4]. The availability of new experimental techniques and tools also allows complex biological processes to be monitored in real time at the single cell level.

The use of engineered gold nanoparticles (Au NPs) in neuroscience has increased considerably over the past decade. Au NPs can easily be bio-conjugated for cell-specific targeting, can be delivered by injection, and match the dimensions of subcellular components, such as those of the cell receptors and ion channels [5]. In the context of stimulation and modulation of neural activity, Au NPs have already been successfully employed for several applications including: enhancement of neurite outgrowth [6,7], modulation of intracellular calcium signaling [8,9], neuron depolarization [10,11], and suppression of neuronal activity [12]. The focus of this review is to provide a critical perspective on the use of Au NPs as an interface to modulate the activity of neuronal tissue. The topics of gene-therapy and cellular uptake and neural toxicity of NPs have been extensively discussed in other recent publications [1,13] and therefore have not been included here.

## 2. Properties of Gold Nanoparticles

### 2.1. Why Gold Nanoparticles?

The integration of Au NPs in neurological research has the potential to find new strategies for diseases that are not currently treatable. This perspective arises from their unique properties, including optical response, chemical and physical stability, relatively low toxicity, and wide range of possible surface functionalizations [13,14]. For example, functionalization with specific ligands allows cellular and molecular specificity, which enables the interaction with target cells and tissues in controlled ways. Thus, Au NPs have been engineered to bind to voltage-gated sodium channels, transient receptor potential vanilloid member 1 (TRPV1) channels, and P2X3 receptor ion channel in dorsal root ganglion neurons [15].

The typical size of Au NPs ranges from approximately 1 to 100 nm, which is comparable to large biological molecules. This favors the interaction with cells, both at the surface and at a fundamental molecular level. In this context, Au NPs have already been used in several biomedical applications, such as biosensing, bioimaging, drug delivery, therapy, and tissue engineering [13]. However, all biological applications require a careful control over biocompatibility. It is well known that some of the most commonly used capping ligands for the fabrication of Au NPs are toxic to cells. A prominent example is the cationic surfactant cetyltrimethylammonium bromide (CTAB), which is commonly used in the preparation of gold nanorods (Au NRs) [16]. CTAB is known to induce cytotoxicity both in vitro [17] and in vivo [18] and to interfere with the surface hydration of the particles [19]. Depositing additional surface coatings has been one of the main strategies to reduce the negative effects caused by residual chemicals used during particle synthesis [20,21]. 

The vast number of applications of Au NPs in biology and medicine is closely related with their unique optical properties. When Au NPs are perturbed by an external light field in the visible or near infrared (NIR) domain, the conduction electrons move away from their equilibrium position, creating a resonant coherent oscillation called the localized surface plasmon resonance (LSPR) [22]. LSPR wavelengths typically fall in the visible to NIR range, with the precise position depending on the particle morphology, interparticle distance, and refractive index of the surrounding medium [23]. For many biological applications, the plasmon absorption peak is selected to match the transparency window of biological tissues (600–1200 nm), meaning that NRs, nanoshells, nanostars, and nanocages appear to be the most suitable morphologies [24]. Despite this, to date only NRs and nanospheres (NSs) have been used for modulation of neuronal activity (see Table 1). Au NRs have proven to be particularly useful, as their resonance wavelength can be tuned by modification of the NR aspect ratio. In addition, they possess two distinct plasmon excitation bands corresponding to the excitation of the short and long axes of the NRs [25]. 

Au NPs also have several attractive features as “high precision” photothermal agents for in vivo neural modulation. As a result of their very small size relative to mammalian cells, Au NPs only heat their immediate environment. This allows the overall heat delivery to be reduced, as long as the particles are strategically positioned close to the target cell. It also leads to a reduction in the diffusion path length for cooling. Consequently, Au NP photothermal modulation acts on sub-millisecond timescales (see the following section), which is critical for temporally precise stimulation of neuronal activity. Moreover, accurate targeting of NPs to the neurons, together with removal of excess particles by the circulation of interstitial fluids, allows off-target environmental heating to be minimized. These properties are likely to be critically important for avoiding damage to thermally sensitive tissues and limiting toxicity due to high concentrations of exogenous particles. Table 1 summarizes the main findings that will be described in the following sections.

### 2.2. Dynamics of the Localized Plasmon Resonance

A range of energy conversion processes occur when an Au NP is irradiated by laser light, and it is useful to understand how these give rise to the various phenomena discussed in this review. Au NPs are typically exposed to a laser source in four distinct time regimes: (i) low-energy femtosecond (fs, ultrafast) laser pulses; (ii) high-energy fs laser pulses; (iii) nanosecond (ns) laser pulses; and (iv) continuous irradiation. The irradiation of metal NPs with an fs pulse leads to a rapid increase in electron energy. For low pulse energies, the temperature of the NP lattice rises by only a few tens of degrees (depending on particle size, optical density, and laser irradiance), while for high pulse energies the temperature of the metal can be raised above its melting point [31]. When ns pulses are applied, the energy threshold for the complete melting of the NRs is effectively reduced due to surface diffusion [32]. In the case of continuous laser irradiation, the particles are constantly saturated, thus reducing their absorption efficiency and the overall photothermal energy conversion.

**Figure 1:**
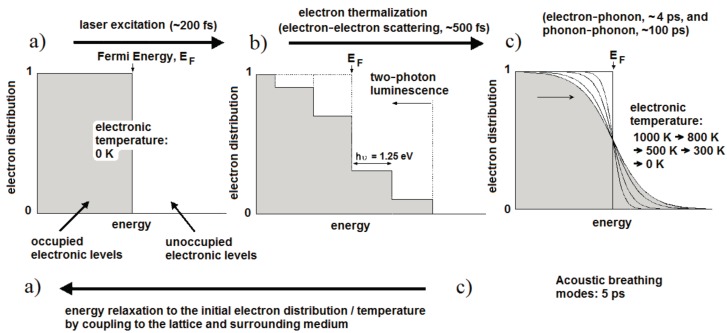
Electron distributions and corresponding timescales of the fundamental processes in laser heating of Au NPs: the initial distribution (**a**) is excited to a non-thermal state (**b**); before relaxing to the Fermi-Dirac distribution (**c**) and finally returning to the initial state once energy has been transferred to the surrounding medium (after Link and El-Sayed [33]).

Figure 1 illustrates the fundamental processes and the timescales that apply at each stage of laser excitation [33]. For convenience, the initial temperature is set to 0 K so that all states up to the Fermi energy are occupied and those above it are unoccupied (Figure 1a). Initially, the light is absorbed by free electrons as shown in Figure 1b, leaving them with a maximum energy equal to the photon energy (1.25 eV for 990 nm light). In the case of fs excitation with high peak intensity, interband transitions can occur from the *d*-band and some excited electrons may undergo a second excitation (Figure 1b). Holes in the low-lying *d* band will recombine with electrons on the tens of fs timescale, resulting in light emission at both longer and shorter wavelengths than the excitation. These are described as single-photon and two-photon luminescence processes. The luminescence efficiency is enhanced by orders of magnitude through coupling of the incoming and outgoing fields to the surface plasmon resonance [33]. The remaining electrons form a non-thermal electron distribution with regard to Fermi-Dirac statistics. These electrons relax into a Fermi-Dirac distribution through electron-electron collisions within a few hundred fs; in this case the electron distribution corresponds to an electron temperature of 1000 K (Figure 1c). The hot electrons proceed to transfer energy into the metal lattice through electron-phonon coupling with a lifetime of about 4 ps, whereupon slower phonon-phonon interactions (~100 ps) transfer energy into the surrounding medium.

Once the excitation is removed, heat conduction into the surrounding medium will lead to the electron gas cooling via the curves shown in Figure 1c, until the system returns to initial state in Figure 1a. If the laser pulse energy is sufficiently high, particle melting (*T*_m_ ≈ 1337 K in gold) and explosive boiling can occur and may be observed in an aqueous medium. Computational modeling of the thermal relaxation process has shown that water at the surface of a 48 × 14 nm^2^ Au NR reaches the critical point (*T*_c_ = 647 K) for laser pulses of 250 fs and an average fluence above about 0.47 mJ·cm^−2^ [31]. Particle reshaping appears to start just above this energy, possibly due to reduced heat dissipation within the gas bubble. For fluences below this level, the temperature across the metal–water boundary typically equilibrates within about 1 ns, with a heated zone of some tens of nanometers [31,34]. Interestingly, Ekici et al. [31] found that exposure to a 1 µs stream of 250 fs pulses at 80 MHz (i.e., 80 laser pulses), generated an overall rise of only 3 K in the water at the particle surface, and this rise was attained during the first few pulses. Larger temperature rises of tens of K can be achieved with continuous wave laser exposure at fluxes of 10^3^–10^5^ W·cm^−2^ and in NP clusters [34]. 

Interestingly, the rapid heating of the lattice generated by fs laser pulses also leads to the impulsive excitation of low frequency acoustic breathing modes of the Au particles (electron–phonon coupling) [35]. The volume of the NPs increases and decreases with a period of about 4–5 ps, which in turn leads to a periodic change in the free-electron density and thus an observable oscillation in the transient absorption [33]. The frequency of these acoustic modes is inversely proportional to the particle radius. A further consequence of the rapid heating is laser-induced reshaping, if the temperature of the NP lattice reaches the melting temperature of Au (pulse energies ~1–10 mJ·cm^−2^). In spherical NPs, the melting may remain unnoticed, but it has been shown that NRs melt into NSs as the most thermodynamically favorable shape within a transformation time of at least 30 ps [36]. This leads to significant bleaching of the longitudinal absorption mode [37]. At higher laser fluences fragmentation of the NPs may also occur, either through vaporization [38] or through ejection of photoelectrons and subsequent electrostatic fragmentation [39]. Although the risk of particle reshaping is reduced when longer laser pulses are applied, it has been shown that ns pulses can produce many partially melted particles, where the shape remains cylindrical but with a rounded mid-section [40]. Depositing additional surface coatings could be a strategy to improve the photothermal stability under ns laser pulse irradiation [41].

## 3. Peripheral Nerve Regeneration

The primary function of a peripheral nerve is to transmit signals from the CNS to the rest of the body, or to convey sensory information from the rest of the body to the CNS. In the case of injury or a health disorder, this pathway can be partially or totally disrupted, resulting in pain, loss of sensation, reduced muscular strength, poor coordination, atrophy, or complete paralysis. Even if peripheral nerves have the capacity of spontaneously regenerating following traumatic injuries, a clinical operation must be performed in case of a complete nerve transaction. Current clinical strategies include autografts, allografts, and nerve guides, yet the maximum regeneration distance is limited to 25 mm [3]. Researchers are currently focused on finding new methods and materials to improve this nerve regeneration distance. Even though the process of neural regeneration is well-known, nerve regeneration following injury remains a great challenge for neuroscientists and neurologists. The process involves outgrowth of neuronal branches (neurites) from the cell body. The neurites elongate, bifurcate, and connect to neighboring neurons to form an electrically functional network. Typically, one of the neurites differentiates into an axon, while the others either turn into dendrites or fail to become functional and retract [42]. 

**Figure 2:**
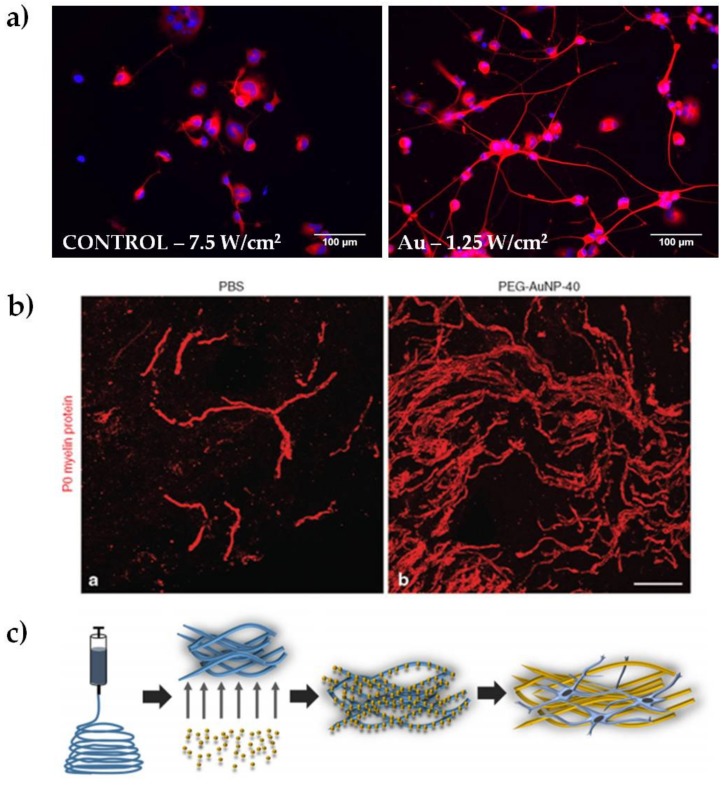
Representative results of Au NPs for peripheral nerve regeneration. (**a**) Examples of epifluorescence images of NG108-15 neuronal cells cultured alone or with Au NRs and exposed to different laser irradiances, as indicated in each panel. Cells were marked for β-III tubulin (in red) and DAPI (in blue, reproduced with permission from [49]); (**b**) Spontaneous remyelination by Schwann cells (myelin marker P0, in red) was enhanced in mice treated with polyethylene glycol-coated Au NPs (reproduced with permission from [7]); (**c**) schematic representation of electrospun nanofibers doped with 10 nm Au NPs (reproduced with permission from [27]).

In our laboratory, we discovered that the heat released by plasmon excitation of Au NRs can be used to stimulate neurite outgrowth in NG108-15 neuronal cells (Figure 2a). The greatest outgrowth was observed after irradiating the endocytosed particles with the highest laser dose (7.5 W/cm^2^), obtaining an average increase in neurite length of almost 36% compared to the non-irradiated sample [6]. We hypothesized that the mechanism underlying the outgrowth involves the activation of one or more transcription factors, supporting previous studies on iron oxide nanoparticles. Indeed, Kim and colleagues performed gene expression analysis in PC12 cells, observing changes in genes related to the cytoskeleton, signaling molecules, receptors for growth hormones, and ion channels [43]. These genes are known to be involved in neuronal differentiation [42]. Papastefanaki et al. used PEG-coated Au NPs after mouse spinal cord injury, showing hind limb motor recovery, attenuation of microglial response, enhanced motor neuron protection, and increased remyelination eight weeks after treatment (Figure 2b) [7]. In a different approach, Bhang and coworkers doped spherical Au NPs with manganese, which allows pH-triggered released of manganese ions after the endocytosis of the particles. They observed neurite outgrowth 24 hours after treatment, showing an increase of roughly 70% compared to control samples. They speculated that changes in intracellular signaling pathways were responsible for the outgrowth increase [26].

Au NPs were also used for integration into nanocomposite nerve conduits. Recently Baranes et al. reported a nerve guide fabricated with electrospun nanofibers doped with 10 nm Au NPs (shown schematically in Figure 2c). The scaffolds encouraged a longer outgrowth of the neurites in primary neurons of the medicinal leech, preferring axonal elongation over the formation of complex networks [27]. Similarly, Das and coworkers reported on a nerve guide fabricated by adsorbing Au NPs onto silk fibers. This nano-hybrid material was successfully tested in a neurotmesis grade injury (complete axonal loss and conduction failure) of a sciatic nerve of Sprague-Dawley rats over a period of 18 months. The nano-composites were found to promote adhesion and proliferation of Schwann cells in vitro and did not elicit any toxic or immunogenic responses in vivo [28]. Lin and colleagues tested chitosan-AuNP microgrooved nerve conduits both in vitro and in vivo. The results showed that the conduits preseeded with primary neuronal stem cells were able to support regeneration of the sciatic nerve better than the controls [29]. Taken together, these studies clearly show that neural regeneration is also influenced by the mechanical support of the guides. Nanoparticle-doped scaffolds open up new strategies to combine bio-materials and nanoparticles for providing physical and/or bioactive environments for neural regeneration. There is also potential to combine the electrical properties of Au NP and bio-materials to promote peripheral nerve elongation [44].

Although the interest in Au NPs for applications in nerve regeneration is expanding, in vivo studies are still limited by a lack of knowledge about the consequences of nanomaterials on intracellular pathways and inflammatory responses. It is known that a high concentration of metal nanoparticles in living organisms can cause cell oxidative stress and reactive oxygen species production, leading to other serious cellular dysfunctions, such as inflammation, cell membrane disruption, DNA damage, cancer, or apoptosis [45]. Söderstjerna et al. recorded a significantly higher number of apoptotic and oxidatively-stressed cells after exposing Au NPs in a primary tissue model of the mouse retina [46]. In our laboratory, we detected a significant oxidative stress increase after exposing NG108-15 neuronal cells to Au NRs for one hour [47]. This result confirmed a previously published report showing oxidative stress generated in the rat brain [48]. Au NPs have also been observed to cause a significant decrease in the levels of dopamine and serotonin in vivo [48]. Moreover, Au NPs have been imaged not only intracellularly, but also intranuclearly, raising questions of whether these nanomaterials can cause DNA damage and/or alter gene expression [46]. However, these effects can generally be minimized by reducing the concentration of NPs and using particles larger than about 15 nm [25].

## 4. Modulation of Nerve Electrical Activity

**Figure 3:**
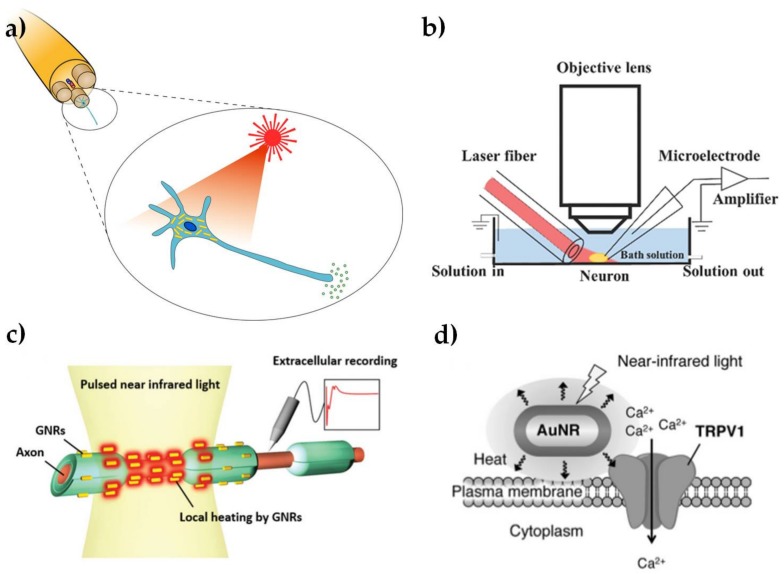
Summary illustration of Au NPs for modulation of nerve electrical activity. (**a**) Schematic representation of laser-induced activation of cells incubated with Au NPs (reproduced with permission from [73]); (**b**) Schematic representation of a whole-cell patch clamp recording for optically-stimulated neurons (reproduced with permission from [10]); (**c**) Schematic representation of optically-stimulated rat sciatic nerves injected with Au NRs (reproduced with permission from [11]); (**d**) Schematic representation of localized heating of TRPV1 channels with NIR excitation of Au NRs (reproduced with permission from [9]).

The use of light to modulate the electrical activity of neuronal cells, as shown schematically in Figure 3a, has attracted growing interest, due to the potential for less invasive neuronal interfaces, improved spatial resolution of stimulation and avoiding electrical artifacts in associated neural recordings [50,51]. The potential to use Au NPs as an exogenous light absorber in neural stimulation appears to have been first identified by [52], but we are not aware of any published demonstration by these workers, who have subsequently focused on the use of black photo-absorbers of ~6 μm diameter [53]. The initial suggestion was based on an analogy with infrared neural stimulation [54], where pulsed laser wavelengths in the range of approximately 1–6 μm have been used to stimulate action potentials in neurons. The primary mechanism in infrared neural stimulation appears to be the transient heating associated with absorption of light by water in the tissue [55,56]. However, water absorption also limits the penetration depth of the infrared light to a few hundred microns [57], while cumulative heating effects tend to limit the stimulation site density and maximum repetition rates [58]. As discussed above, Au NRs allow highly localized photothermal heating through the absorption of wavelengths in the water transmission window from 600 to 1200 nm.

Yong et al. [10] first confirmed that Au NRs can be used to stimulate cultured rat primary auditory neurons with near-infrared (780 nm) illumination. The laser-induced cell electrical activity was observed using whole cell patch clamp electrophysiology, as shown in Figure 3b. The open patch technique was used to show that action potentials were associated with transient temperature increases of about 6 °C. The NRs were endocytosed by the neurons after 15–17 h incubation, as shown by dark field microspectroscopy. This work was soon followed by a demonstration that Au NRs could be used to elicit compound action potentials in the rat sciatic nerve in vivo (shown schematically in Figure 3c) [11]. The NRs with peak absorption at 977 nm were introduced to the nerve bundle by micro-injection. Subsequent TEM analysis of fixed cross-sectional slices showed Au NRs located near the surface of the axon plasma membrane. In contrast, Yoo et al. [12] found that Au NRs inhibited neural activity in networks of primary cultured hippocampal neurons. This inhibitory effect was associated with longer laser exposures (1–30 min) and the NPs were coated with positively-charged amine-terminated polyethylene glycol, which may have had an increased affinity to attach to the cell membrane. The increased exposure time led to a sustained temperature rise of as much as 10 °C at the plasma membrane. It is well known that a sustained increase in environmental temperature can have an inhibitory effect on neural activity [59] and similar effects have been observed in infrared neural stimulation [60]. 

Although the detailed mechanism is not yet understood, the broad principles for these effects do indeed appear to be analogous to infrared neural stimulation. In particular, the local increase in temperature due to plasmonic heating produces a change in the electrical capacitance of the plasma membrane [10], in agreement with the observations of Shapiro et al. [55]. In isolation, these changes in cell capacitance are unlikely to act as an excitatory stimulus, except in the most voltage sensitive cells [61,62]. However, infrared-induced temperature changes have also been shown to modulate the responses of voltage- and temperature-sensitive (TRPV) ion channels [63,64], as shown in Figure 3d. Modulation of Ca^2+^ dynamics in the soma may also be involved [8], again in analogy with effects observed for infrared neural stimulation [65]. Nakatsuji et al. [9] have subsequently confirmed that laser heating of Au NRs causes Ca^2+^ influx by TRPV1 activation. The surface chemistry of the NPs was modified with a cationic lipoprotein for non-cytotoxic targeting of the plasma membrane and the effect was also demonstrated in primary cultured dorsal root ganglion cells from wild type mice. 

Finally, it is has been shown that relatively high levels of infrared laser exposure can lead to nanoporation of the cell membrane, with concomitant Ca^2+^ uptake and cellular swelling [66]. Once again, similar disruptions of the cell membrane have been observed in the presence of Au NPs, across a wide range from continuous wave to fs laser pulse lengths [67,68]. However, it appears unlikely that this level of disruption is generated by the relatively modest laser irradiances involved in NR-mediated neural modulation [10,11,12]. On the other hand, it is known that nanoscale heating with Au NSs can induce a gel-fluid phase transition in phospholipid giant unilamellar vesicles [69]. Subsequent studies have shown that membrane conductance can be controlled by plasmonic heating of single Au NPs over periods of several seconds, without a phase transition or nanopore formation. It was proposed that this effect is due to an increase in phospholipid mobility with increasing temperature and was observed in both artificial lipid bilayers formed on a planar patch clamp system and in HEK293 cells that lack temperature-sensitive ion channels [70]. Similar experiments with artificial membranes composed of asolectin have interpreted the transient current during initial heating in terms of capacitance changes, with a single NP producing a capacitive current of 0.75 pA under irradiance of 18 kW/cm^2^ for ~1 ms [15]. Further work is needed to clarify the relative importance of these various contributions, their relationship to the ensuing biochemical pathways, and any long term deleterious effects that may arise.

Recently, it has been shown that less than 1 ms of NIR stimulation combined with Au NRs reliably produces strong Ca^2+^ transients in astrocytes [30]. While this interaction may help to facilitate minimally invasive studies of astrocyte function, it also points to the importance of targeting NPs to specific locations in the tissue. Eom et al. [30] targeted the astrocyte surface with biotinylated anti-thymocyte antigen-1 antibody and streptavidin-coated Au NRs. Carvalho-de-Souza et al. have demonstrated that Au NSs can be conjugated with functional groups that target voltage-gated sodium, TRPV1, and P2X3 ion channels, all of which are known to be expressed in the membrane of dorsal root ganglion neurons [15]. In each case, it was found that the Au NPs bound to the cultured neurons without impeding their excitatory capability and generated optically-evoked action potentials at relatively low NP concentrations. In comparison, unconjugated Au NPs required higher concentrations to support optical stimulation and were readily washed out on solution exchange. Targeting membrane receptors is an important approach, as cells of different sensory specialization can express very different profiles of membrane receptors. However, to our knowledge, neuronal selectivity has yet to be demonstrated in mixed cultures or in vivo. Interestingly, it has been found that organically-modified silica (ORMASIL) nanoparticles are preferentially taken up by neurons in vivo [71], but it is not yet clear what mechanism is involved and whether it can be extended to Au NPs.

The work reviewed above demonstrates the potential to improve the stimulation efficiency and increase the penetration depth of infrared neural stimulation by labeling nerves with Au NRs. Although this review has focused on applications of Au NPs in neural stimulation, a range of nanomaterials with optical, electrical, magnetic, mechanical, and chemical sensitivities have also attracted attention in this context, as recently reviewed by Colombo et al. [72].

## 5. Outlook

Applications of nanotechnology in basic and clinical neuroscience are in the early stages of development, partly because of the complexities associated with neural cells and the CNS. Many of the biochemical, cellular, and genomic mechanisms of neural regeneration, modulation, and nanomaterial-tissue interactions are still not well understood. The challenges are numerous, but the impact that NPs can have on understanding the physiology and the pathology of the nervous system and how we can intervene at a molecular level is significant. From a technical point of view, NPs need to be engineered to obtain a greater cellular specificity, multiple induced physiological functions (such as targeting of multiple cell receptors, ligands, or synapses), and minimal side effects [74]. The current consensus [25] is that Au NPs are larger than about 15 nm are less toxic than smaller particles, and that the primary cytotoxicity in Au NRs is associated with CTAB, the cationic capping ligand that is typically used in the preparation. The toxicity issues can be largely overcome by uniformly coating the NP surfaces with biocompatible polymers or molecules [13,47] and carefully targeting them to the required location. However, there remains a need to clarify the long term effects of NPs on gene expression, activation of specific intracellular pathways, neurotransmitter release, and cellular inflammation.

In terms of electrical activity, careful studies are needed to investigate the influence of NPs on single ion channels and ionic currents. Carvalho-de-Souza et al. [15] have shown that Au NPs can be targeted to neuronal membrane receptors without hindering their excitatory functioning and resisting washout for periods of more than 30 min. However, the Au NP conjugates will have a limited lifetime due to the natural turnover of membrane proteins, and clearance and/or degradation of the NPs. Repeated treatment may lead to NP accumulation. It has been observed that intracellular Au NPs can increase neuronal excitability and aggravate seizure activation in hippocampal tissue, therefore suggesting that intracellular NPs might alter neuronal functions and cause hyper-excitability under pathological conditions [75]. 

Au NPs have attracted attention in a wide range of medical, therapeutic, and technological contexts [13,24]. For example, Boulais et al. [76] review the interaction of short and ultrashort laser pulses with plasmonic NPs for the purpose of destroying, modifying or manipulating molecular, sub-cellular, and cellular structures. In these applications, heating, low-density plasma generation, pressure wave release, and formation of vapor bubbles can be used to disrupt cells for drug delivery and cell transfection, typically by nanoporation. Au NPs can also be used as exogenous contrast agents for photoacoustic imaging [77]. Considering the steadily growing knowledge and the impressive versatility demonstrated by Au NPs in this wide range of applications, we confidently expect further progress and innovation in the field of neuromodulation.

## Figures and Tables

**Table 1:** Summary of Au NP characteristics for modulation of neural activity. Plasmon peaks have only been indicated when relevant to the study.

Shape	Size	Plasmon Peak	Functionalization	Applications	Observed Effects
Nanorods	48.6 nm × 13.8 nm	780 nm	Poly(4-styrenesulfonic acid), silica	Peripheral nerve regeneration	Increased neurite length [6]
Nanospheres	40 nm	-	Polyethylene glycol (PEG)	Peripheral nerve regeneration	Hind limb motor recovery, attenuation of microglial response, enhanced motor neuron protection, increased remyelination [7]
Nanospheres	8.6 nm	-	Manganese-doped	Peripheral nerve regeneration	Increased neurite length [26]
Nanospheres	10 nm	-	-	Integration into nerve conduits	Increased neurite length [27]
Nanospheres	2–22 nm	-	-	Integration into nerve conduits	Promote adhesion and proliferation of Schwann cells [28]
Nanospheres	5 nm	-	Chitosan	Integration into nerve conduits	Regeneration of the sciatic nerve [29]
Nanorods	Aspect ratio 3.4	780 nm	Silica	Modulation of electrical activity	Action potentials in primary auditory neurons [10]
Nanorods	80.4 nm × 15.3 nm	977 nm	-	Modulation of electrical activity	Action potentials in rat sciatic nerves in vivo [11]
Nanorods	71.3 nm × 18.5 nm	785 nm	Amine-terminated PEG	Modulation of electrical activity	Inhibition of neural activity in primary hippocampal neurons [12]
Nanospheres	20 nm	532 nm	Functional groups that target voltage-gated sodium, TRPV1 and P2X3 ion channels	Modulation of electrical activity	Action potentials in dorsal root ganglion cells [15]
Nanorods	48.6 nm × 13.8 nm	780 nm	Poly(4-styrenesulfonic acid)	Modulation of Ca^2+^ dynamics	Intracellular Ca^2+^ transients [8]
Nanorods	60.0 nm × 15.0 nm	780 nm	Cationic protein/lipid complex	Modulation of Ca^2+^ dynamics	Ca^2+^ influx by TRPV1 activation [29]
Nanorods	82.9 nm × 13.4 nm	982 nm	Streptavidin	Modulation of Ca^2+^ dynamics	Ca^2+^ transients in astrocytes [30]
